# The Marker State Space (MSS) Method for Classifying Clinical Samples

**DOI:** 10.1371/journal.pone.0065905

**Published:** 2013-06-04

**Authors:** Brian P. Fallon, Bryan Curnutte, Kevin A. Maupin, Katie Partyka, Sunguk Choi, Randall E. Brand, Christopher J. Langmead, Waibhav Tembe, Brian B. Haab

**Affiliations:** 1 Laboratory of Cancer Immunodiagnostics, Van Andel Institute, Grand Rapids, Michigan, United States of America; 2 Carnegie Mellon University, Pittsburgh, Pennsylvania, United States of America; 3 University of Pittsburgh Medical Center, Pittsburgh, Pennsylvania, United States of America; 4 Translational Genomics Research Institute, Phoenix, Arizona, United States of America; Queen Elizabeth Hospital, Hong Kong

## Abstract

The development of accurate clinical biomarkers has been challenging in part due to the diversity between patients and diseases. One approach to account for the diversity is to use multiple markers to classify patients, based on the concept that each individual marker contributes information from its respective subclass of patients. Here we present a new strategy for developing biomarker panels that accounts for completely distinct patient subclasses. Marker State Space (MSS) defines “marker states” based on all possible patterns of high and low values among a panel of markers. Each marker state is defined as either a case state or a control state, and a sample is classified as case or control based on the state it occupies. MSS was used to define multi-marker panels that were robust in cross validation and training-set/test-set analyses and that yielded similar classification accuracy to several other classification algorithms. A three-marker panel for discriminating pancreatic cancer patients from control subjects revealed subclasses of patients based on distinct marker states. MSS provides a straightforward approach for modeling highly divergent subclasses of patients, which may be adaptable for diverse applications.

## Introduction

The development of accurate biomarkers is increasingly important with the growth of molecular and personalized medicine [1]. Biomarkers are needed for diverse areas such as risk assessment, early detection, differential diagnosis, disease staging and prognostication, treatment selection, and treatment monitoring [1], [2]. The development of cancer drugs that are targeted to specific molecules is now generally accompanied by the development of companion diagnostic biomarkers that can select the patients most likely to benefit from the drug or can monitor the efficacy of the drug [3], [4]. A biomarker can consist of any measurable physical quantity, such as cell counts or nodal status, but modern efforts at biomarker development are mainly focused on specific molecules within the classes of DNA, RNA, proteins, carbohydrates, lipids, and metabolites. Molecular entities may provide more objectivity and accuracy than traditional modes of evaluation, since specific molecules may be functionally involved in the mechanism of the pathology.

A challenge in the development of molecular biomarkers arises from the diversity between people in the molecules that are present in disease. Because of variation between people in genetics, environment, and disease status, any single molecular biomarker usually does not provide an accurate diagnosis for every individual. An example is prostate specific antigen, which is routinely used to screen for prostate cancer, but is frequently elevated in non-cancerous conditions and not elevated in some cancers [5]. A common approach for addressing this challenge is to use multiple molecular biomarkers together in a single biomarker panel [6]. The rationale for that approach is that the diversity between people can be accounted for through multiple biomarkers, each of which contributes information for a particular subset of the population. The potential for improved accuracy of combination biomarkers over single biomarkers has been demonstrated in numerous cases.

A critical consideration in the development of combination biomarkers is how individual markers should be selected and what rules should be used in bringing them together. The concept of using multiple factors to model and predict classes has been extensively studied in many different fields, and many systems have been developed. In the field of biomarker discovery, frequently used techniques are recursive partitioning, linear discriminant analysis, and logistic regression [6], [7]. All have been used to search for combinations of biomarkers for various types of cancer, including lung [8], [9], [10], prostate [8], [11], breast [8], colorectal [8], and gastric [8] cancers. Recursive partitioning provides a straightforward classification method and can function despite missing data values. Additionally, though the selection of markers can be unstable with certain data sets [12], techniques exist for optimizing this procedure [13], [14]. Linear discriminant analysis is robust but is limited by its assumption of a normal data distribution and equal class covariance [15], which are difficult requirements to meet in a clinical setting. Logistic regression is able to capture the scalar nature of many factors [16] but may not accurately model sharp distinctions between patient subclasses. Each has its own strengths and limitations when applied to the development of a biomarker panel [16].

Here we present an alternative method for developing biomarker panels, called Marker State Space (MSS). MSS is designed with the recognition that subclasses of patients can have completely distinct molecular characteristics, as observed with several cancers [17], [18], [19]. To account for divergent subgroups, MSS allows distinct “states” to exist within either the cases (patients with disease) or the controls (unaffected subjects). A state refers to the pattern of high and low values among a set of markers, and the state space is the set of all possible patterns. By defining certain states as case states and others as control states, all subclasses of molecular diversity are encompassed within a set of markers. This method is distinguished from other methods of classifier development by its systematic categorization of all possible patterns of biomarker levels using binary (high or low) values.

The purpose of the present study was to determine if the MSS approach could identify robust multi-marker panels and molecular subclasses of patients. We developed software for identifying biomarker panels using MSS and tested the method in the development of a biomarker panel for differentiating patients with pancreatic cancer from those with a benign condition of the pancreas such as pancreatitis. Such biomarkers are needed because of the clinical similarity between those conditions and the critical need to make accurate diagnoses as early as possible [20]. The marker data were acquired using a method for detecting glycan levels on the proteins captured by the antibody microarrays [21], [22], [23], which has the potential for enhanced biomarker performance relative to conventional protein detection [22], [24]. We demonstrate that biomarker panels developed using MSS are robust in cross validation and training-set/test-set analyses and that MSS provides a novel approach for identifying patient subclasses based on marker states.

## Experimental Section

### Biological reagents

The antibodies and proteins were purchased from various sources (Table S1). The antibodies were purified by dialysis (Slide-A-Lyzer, Pierce Biotechnology, Rockford, IL) against PBS buffer followed by ultracentrifugation. The concentration of each antibody was adjusted to 250 µg/ml prior to printing. The integrity and purity of each antibody was confirmed by SDS-PAGE under reducing and non-reducing conditions. The manufacturers characterized the specificities and optimal applications of most of the antibodies, and we performed Western blot and dilution series analyses for a subset of them [25], [26]. Antibody biotinylation was performed using EZ-Link sulfo-NHS-LC-biotin (sulfosuccinimidyl-6-(biotinamido) hexanoate (Pierce Biotechnology, Rockford, IL).

### Plasma samples

Plasma samples (using EDTA as the anti-coagulant) from the University of Pittsburgh School of Medicine were collected from pancreatic cancer, pancreatitis and healthy subjects. Early-stage cancer was defined as stages I and II, and late-stage cancer was defined as stages III and IV. The pancreatitis patients were a mixture of chronic and acute. The control subjects were healthy with no evidence of pancreatic, biliary or liver disease. The samples at each site were collected using a standard operating procedure based on the serum and plasma protocols from the Early Detection Research Network. All samples were stored at −80°C and sent frozen on dry ice. Each aliquot had been thawed no more than three times before use.

### Ethics statement

All sample collection and research was conducted under protocols approved by the Institutional Review Boards at Evanston Northwestern Healthcare, the University of Pittsburgh School of Medicine, and the Van Andel Research Institute. Written, informed consent was obtained from all participants in the study.

### Antibody-array assays

Antibody microarrays were prepared to detect glycan levels on captured proteins and glycans. A piezoelectric non-contact printer (2470 Arrayer, Aushon Biosystems, Billerica, MA) was used to spot ∼350 pl of each antibody solution on the surfaces of ultrathin nitrocellulose-coated glass microscope slides (PATH slides, GenTel Biosciences, Madison, WI). Forty-eight identical arrays were printed on each slide with each array consisting of 16–48 different antibodies as well as control immunoglobulins from several species printed in triplicate. A wax border was imprinted around each of the arrays to define hydrophobic boundaries (SlideImprinter, The Gel Co., San Francisco, CA). The printed slides were stored at 4 °C in a desiccated, vacuum-sealed slide box until use.

Antibody-array assays were performed to measure glycan levels on either captured proteins or captured glycans (Fig. S1). Serum samples were diluted with PBS buffer containing 0.1% Brij, 0.1% Tween 20, and 50 µg/mL protease inhibitor mixture (Roche Applied Science, Indianapolis, IN). A blocking solution consisting of final concentrations of 400 µg/mL goat, mouse, and sheep IgG; 400 µg/mL chicken IgY; and 800 µg/mL rabbit IgG was included in each serum or plasma sample to reduce nonspecific binding to the printed antibodies. Slides were blocked with 7μL PBS with 0.5% Tween 20 (PBST0.5) and 1% BSA for 1 hr at room temperature with gentle shaking. The slides were washed in three changes of PBST0.5 for 3 min each with rocking. Six microliters of each sample solution were incubated on each array overnight at 4°C. The slides were washed in three changes of PBST0.1 for 3 min each with rocking. Captured antigens were detected with biotinylated antibodies at a concentration of 3 µg/mL followed by incubation with 2 µg/mL streptavidin-phycoerythrin (Roche Applied Science, Indianapolis, IN) using incubation and wash conditions as above. The slides were scanned for fluorescence emission at 532 nm using a microarray scanner (LS Reloaded, Tecan, Männedorf, Switzerland). All arrays were scanned concurrently at a single laser power and detector PMT gain setting.

Image data were quantified using GenePix Pro 5.1 (Molecular Devices, Sunnyvale, CA). The net fluorescence signal was calculated by subtracting the median local background surrounding each spot from the median intensity of the corresponding spot. The signal intensities from replicate antibody measurements within the same array were averaged (geometric mean). Antibodies were removed from subsequent analysis that gave low signals over most of the samples, defined as <10% of samples giving signal at least two-fold higher than the signal in the negative control array (incubated with PBS instead of plasma).

### Marker State Space program

The exhaustive marker search and selection algorithm has been implemented in C/C++ using standard libraries and tested on Linux and Mac OS platforms. The program exhaustively analyzes all combinations of three markers, and all possible combinations of thresholds for each marker within each group of three, to determine a sensitivity and specificity for each combination. The user specifies the step size for scanning through the thresholds and the minimum specificity and sensitivity that is required for a marker combination to be included in an output file. The program also performs 10-fold cross validation and returns results from each of the 10 splits of the analysis. Details of this procedure are provided below. The software is available on request to the authors.

### Details of the marker selection program and procedures

The program first carries out a training process on all of the sample data (“full train”) for all possible combinations of three markers. For each marker, the program tests all possible thresholds within that marker’s range of data values. The level of detail with which this process is carried out can be customized using the “threshold step.” This variable sets the interval between successive thresholds in the scanning process. For example, if a step is set at 0.5, a marker with log fluorescence values ranging from 1.25 to 3.25 will be tested with the thresholds of 1, 1.5, 2, 2.5, 3, and 3.5. The threshold step can be increased to optimize the speed of the program (as might be desired with a very large set of data) or it can be decreased to achieve a greater level of detail in the results.

Once finished with the full train the program moves on to a 10-fold cross validation testing process. The samples are randomly divided into ten groups, or “splits,” of equal (or as close to equal as possible) number. Data for nine of the ten groups are compiled and used for training, examining all possible combinations of three markers at all thresholds (using the same thresholds for each marker that were used in the full train). The panels meeting or exceeding a user-defined minimum performance are subsequently tested against the remaining tenth split, where their accuracies are recorded. This is repeated ten times, with each split serving as the test set exactly one time.

Upon completion the program generates several text files; a series of files for each split and for the full train. Almost all of the information contained in these is compiled into two summary files. A “final report” lists every panel generated (that met sensitivity/specificity cut-offs), along with its sensitivity/specificity in the full train, accuracy in each split (for any splits in which a panel did not meet minimum performance N/A is reported in place of accuracy), and average accuracy across the splits. A “detailed” file contains the classification rules, with a breakdown of the samples populating each, for every panel that met the sensitivity/specificity cut-offs. The remaining output files contain more details regarding the ten splits, such as the samples used in each.

The strategy we developed for selecting the most robust marker panel begins by sorting through the “final report,” opened as a Microsoft Excel spreadsheet. The list of panels is trimmed to include all the panels that tie for first or second best performance in any of the ten splits. Using this trimmed list, the number of appearances of each unique combination of three markers is counted. This count represents the number of different thresholds at which each set of three markers appeared as a top panel. The ability of a panel to achieve high accuracy at several unique sets of thresholds is an indicator of robustness, or insensitivity to slight variations in sample data. A high average accuracy in the ten splits is also an indicator of robustness, and so an average of each panel’s average in the splits is calculated. Count and average accuracy in the splits are compiled for each combination of three markers, and the marker panel that performs best between the two categories is selected for further validation.

## Results

### The Marker State Space method

**Figure 1:**
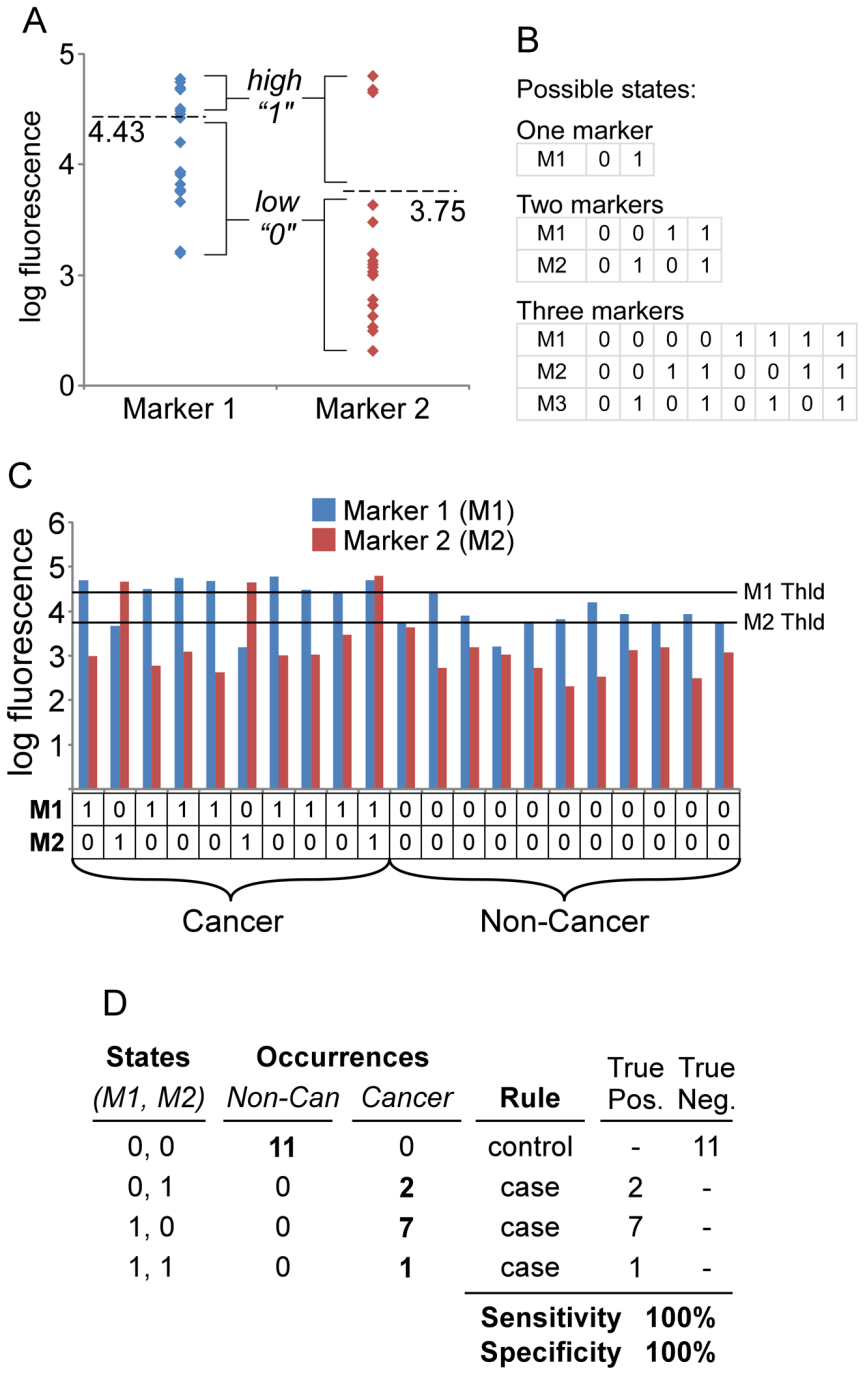
Assigning patient classes and classifying marker states. (A) Thresholding the data. Representative data for 21 samples are presented, in which each point represents a patient sample measurement for Marker 1 (left) or Marker 2 (right). A threshold (dashed line) was applied to each marker. Values above the threshold are converted to 1 and values below the threshold are converted to 0. (B) Possible states. Each column represents a unique state for panels of 1, 2, or 3 markers. (C) Determining marker states for each patient. The data from both Marker 1 and Marker 2 are presented for each of the 21 patients, along with their respective thresholds (horizontal lines). The thresholded data are below the column graph. Each sample has a particular marker state (0,0; 0,1; 1,0; or 1,1). (D) State classification. Each state is classified as either case or control based on whether cancer or non-cancer samples have a greater number of occurrences in that state. The “true positives” are the cancer samples that occupy case states, and the “true negatives” are the non-cancer samples that occupy control states. These values are used to calculate the sensitivity and specificity for the panel.

The Marker State Space (MSS) method operates on a binary system in which each individual marker is either high (1) or low (0), based on a threshold for that marker (Fig. 1A). The state space is the combinations of 1s and 0s that are possible for a certain number of markers. A panel of two markers has four possible states: 0,0; 0,1; 1,0; and 1,1; and a panel of three markers has eight possible states: 0,0,0; 0,0,1; 0,1,0; 0,1,1; 1,0,0; 1,0,1; 1,1,0; and 1,1,1 (Fig. 1B). (Panels with more markers would have 2^n^ possible states, n being the number of markers.) A given sample occupies exactly one state, depending on its pattern of high and low values for each marker. In order to classify samples, each state is designated as either a “case” state or a “control” state. For example, in a two-marker panel, the state 0,0 could indicate control samples, and the states 0,1; 1,0; and 1,1 could indicate case samples (Figs. 1C and 1D).

**Figure 2:**
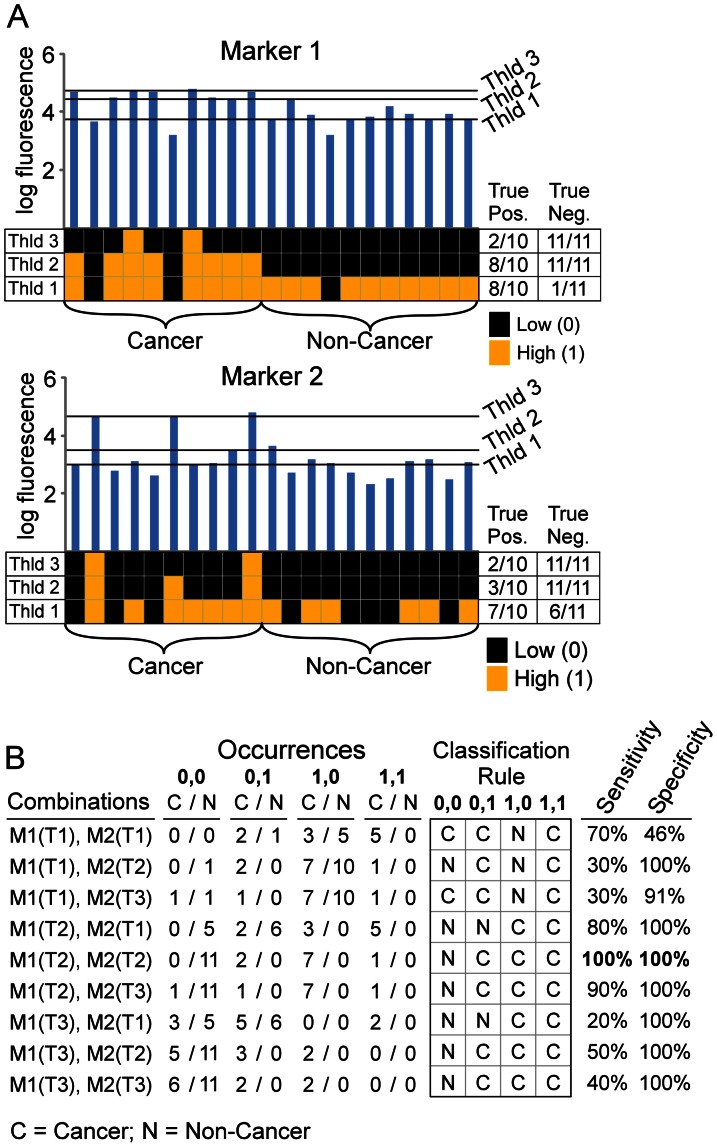
Determining optimal thresholds for a two-marker panel. (A) Scanning thresholds. Three different thresholds are depicted for Marker 1 (left) and Marker 2 (right), with the resulting conversion to 1s and 0s for each threshold, followed by the sensitivities and specificities for each marker at each threshold. (B) Determining the best combination of thresholds. All possible combinations of thresholds were assembled for the two-marker panel, resulting in nine combinations. Based on the results from panel A, the numbers of cancer and non-cancer samples that occupy each state were determined for each combination, from which the sensitivity and specificity could be calculated for each combination. The combination of thresholds giving the best performance (in this case threshold 2 for Marker 1 and threshold 2 for Marker 2) is selected.

The discovery of biomarker panels based on this classification system requires a method for selecting the members of the marker panel, the thresholds for each marker, and the state rules (the designation of which states are cases and which states are controls). These three factors, the markers, the thresholds, and the state rules, are related to each other, so that changes in one might affect the optimal values for the other two. An approach to selecting the thresholds and state rules that best discriminate two groups of samples is illustrated in Figure 2 for two markers. For each individual marker, several test thresholds are applied to convert the data to 1s and 0s (Fig. 2A). To determine which thresholds work best together between the markers, all nine possible combinations could be examined (Fig. 2B). For each of these combinations, we can assign certain states to indicate cases and other states to indicate controls. A simple approach to making those assignments is to count how many case and control samples populate each state, and then make the assignment accordingly (Fig. 2B). For example, if state 0,1 is populated by six control samples and only two case samples, the state would be assigned to indicate controls. Once the assignment is made for each state, all samples in each state are classified according to the assignments. A sensitivity and specificity can be calculated based on how many case and control samples were correctly classified.

The specificities and sensitivities of each of the combinations of thresholds can be compared to determine which combination gave the best discrimination between cases and controls (Fig. 2B). In the example of Fig. 2, the use of threshold 2 for Marker 1 and threshold 2 for Marker 2 gave perfect classification of the cases and controls, but all of the other combinations of thresholds gave some misclassifications.

The next level of selection occurs when data from multiple candidate biomarkers are available. For example, when collecting data from antibody microarrays, measurements from dozens of antibodies might be acquired. To find the combination of markers that work well together, a combinatorial search is required to test the performance of various groupings of markers. For example, a search for a two-marker panel among a dataset containing many different markers could function by first testing Marker 1 with Marker 2, covering all combinations of thresholds, then testing all combinations of thresholds using Marker 1 with Marker 3, next Marker 1 with Marker 4, etc. For a three-marker panel, many more combinations are possible.

Because of the significant computation required to explore the combinations of markers and thresholds that give the best biomarker performance, we developed software to perform that search. The initial version of the program was designed to search for panels of three markers. This choice was made to keep down the computation time and to gain more information about the performance of the method with relatively simple panels. The user may select the step size for scanning through the thresholds for each marker, using linear steps through thresholds over log-transformed data. The user also may select the minimal levels of performance for a marker panel to be included in an output file. Additional details of the operation and output of the software are provided in the methods section.

### MSS applied to the selection of a biomarker panel

The MSS method was applied to the discovery of a plasma biomarker panel that could accurately distinguish cancer patients from pancreatitis patients. Antibody microarray measurements were acquired from 197 plasma samples, comprising 121 from pancreatic cancer patients and 76 from pancreatitis patients. We used a technique in which glycan levels are probed on the proteins captured by antibody microarrays [21], [22], [23], owing to the potential for enhanced biomarker performance relative to conventional protein detection [22], [24]. Each plasma sample was incubated on an array containing 36 different capture antibodies and control antibodies (Table S1), and the captured proteins were probed with an anti-glycan antibody (Fig. S1). The capture antibodies were chosen to target glycoproteins and glycan epitopes that potentially have altered abundance in pancreatic cancer, based on literature sources and our own previous research [22], [27], [28], [29], [30], [31]. Eight different anti-glycan detection antibodies were used on each sample in individual arrays. The detection antibodies targeted the CA 19-9 antigen (a polysaccharide called sialyl-Lewis A) and related glycans such as Lewis blood group structures and glycans from the ABO blood group system. These glycans were chosen to determine whether other specific glycans in additional to the CA 19-9 antigen are elevated in particular cancer patients. Because each combination of capture antibody and detection antibody makes a unique assay, the eight arrays for each sample resulted in 288 (368) total capture-detection pairs and candidate marker assays for each sample.

Before beginning the marker selection process, we culled the data to remove the positive and negative control antibodies and the assays that gave very weak signals over all the samples (see methods section for criteria). A resulting set of 127 capture-detection pairs was used in the subsequent analysis. The raw fluorescence values were log transformed (base 10), which converted the values from the 16-bit range of 0 to 65,535 to a range of 1 to 4.82. The MSS program scanned through thresholds over that range for each marker using a step of 0.2. This step size was selected to balance the competing factors of processing time and detail in the analysis. A comprehensive search of all combinations of markers and all thresholds for each marker uncovered a three-marker panel that had an accuracy (rate of correct calls) of 89.9%, with a sensitivity of 89.3% and a specificity of 90.8%. This performance is similar to that achieved by a variety of other methods for developing multimarker classifiers (Table 1).

**Table 1:** Comparison of performance between methods.

	All Data (197 samples)			10-Fold Cross Validation*		
Method	Accuracy	Sensitivity	Specificity	Accuracy	Sensitivity	Specificity
MSS	89.9%	89.3%	90.8%	84.7%	–	–
SVM	88.8%	88.4%	89.5%	87.3%	87.6%	86.8%
Logistic Regression	87.3%	89.3%	84.2%	87.3%	87.6%	86.8%
Naïve Bayes	82.7%	77.7%	90.8%	82.7%	77.7%	90.8%
Neural Net	87.3%	88.4%	85.5%	85.3%	88.4%	80.3%
K-Nearest Neighbor	90.4%	90.9%	89.5%	85.3%	87.6%	81.6%

	Training Set (131 samples)			Test Set (66 samples)		
Method	Accuracy	Sensitivity	Specificity	Accuracy	Sensitivity	Specificity
MSS	87.8%	88.9%	86.0%	89.4%	85.0%	96.2%
Logistic Regression	86.3%	87.7%	84.0%	92.4%	87.5%	100.0%

* The software did not calculate an average sensitivity and specificity for MSS in 10-fold cross validation because its does not separately calculate those parameters in each cross validation split.

Cross validation is an important tool for estimating the performance of a biomarker panel in future samples. A panel is built from a subset of the available samples and then applied to the remaining samples. That process can be repeated with several different divisions of training sets and test sets. The average of the performance of each biomarker panel on the test sets of samples gives a reliable indicator of the robustness of a panel that can be derived from the existing data. Cross validation was applied to the sample set using the MSS method. Ten-fold cross validation was used, meaning that the samples were divided into ten parts, and for each iteration, a biomarker panel was built from nine parts and applied to the tenth. The average accuracy of panels derived from MSS was 84.7%, only slightly lower than the performance on the entire data (Table 1). The fact that performance held up well in the cross-validation test sets indicates good potential for continued accuracy in future samples. This performance was similar to that achieved by logistic regression (Table 1).

Next we sought to investigate more deeply the performance of a single panel in a training set and a test set. Two thirds of all the available sample data were used for a training set (to develop a panel), and the remaining one third was set aside as a test set (to test the panel). The two sets had equivalent percentages of cases and controls. We used the results from cross validation analysis on the training set to develop a candidate biomarker panel. We reasoned that the most robust panel would have nearly the best performance in each of the 10 iterations in cross validation and that it would give good performance at many different thresholds for each of the markers in the panel. The three-marker panel that showed the best performance by these considerations was CA19-9 (Ab1)-CA19-9 (Ab1), Blood group A-Blood group B, and CA19-9 (Ab2)-CA19-9 (Ab1). (Each marker is defined by a capture antibody and a detection antibody, indicated before and after the dash.) We previously showed that the two CA 19-9 antibodies used here, Ab1 and Ab2, have differing and complementary specificities [32], which provides a molecular understanding of their inclusion together in a biomarker panel.

**Figure 3:**
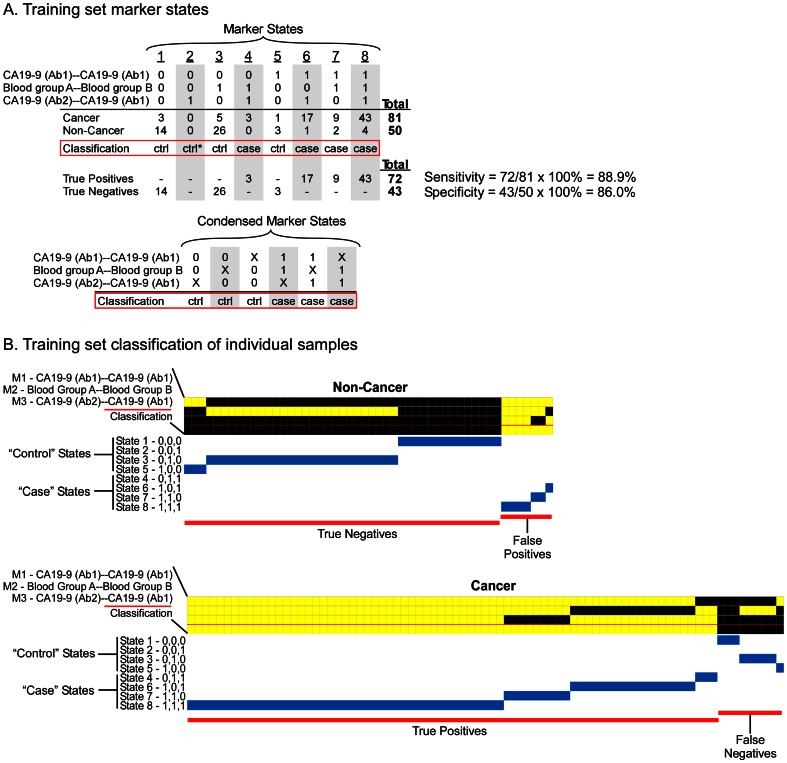
Training set marker states and patient classifications. (A) Training set marker states. The eight possible marker states for the three indicated markers are shown, followed by the numbers of case and control samples in each state and the categorization of each state. *State 2 was unoccupied by categorized as a control state because of similarity to other control states. The lower panel shows condensed marker states, in which X indicates either 0 or 1. (B) Individual sample classifications. Each column represents an individual patient sample, and the first three rows indicate results from the indicated markers. A yellow square indicates the sample was above the threshold for that marker, and black indicates below. The blue lines indicate the state in which each sample was classified.

Before applying this panel to the test set, we used the entire training set data to find the thresholds and state rules that gave the best performance. The panel achieved 88.9% sensitivity, 86.0% specificity, and 87.8% accuracy (Table 1). State 4 (0,1,1), state 6 (1,0,1), state 7 (1,1,0), and state 8 (1,1,1) were mainly occupied by case samples and therefore were classified as case states (Fig. 3A). State 1, state 3, and state 5 were classified as control states. State 2 (0,0,1) was not occupied by any sample but was classified as a control state due to its similarity to the other control states. These state rules can be condensed into a simplified rule that if a sample is elevated in any two or more of the markers, it is called a “case” (Fig. 3A).

**Figure 4:**
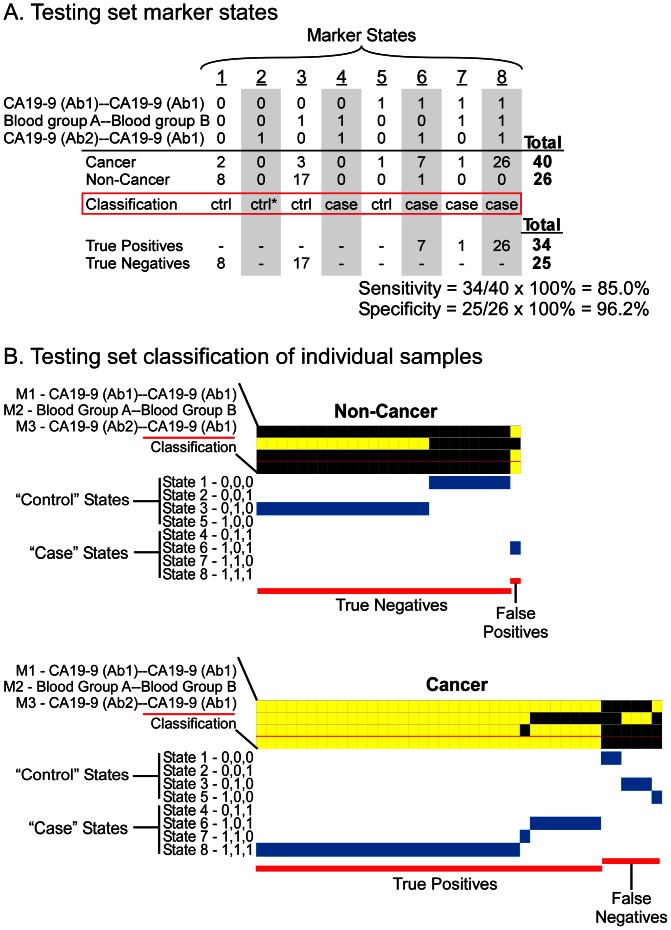
Test set marker states and patient classifications. The same marker panel, thresholds, and classification rules as shown in figure 4 were applied to the one-third of the total samples that were separated as a test set. (A) Occupancy of the marker states in the test set. (B) Individual sample classifications in the test set.

The application of this three-marker panel to the test set achieved sensitivity of 85%, specificity of 96.2%, and accuracy of 89.4% (Fig. 4A). This performance held up well relative to the training set (even slightly improved) and was similar to that achieved by logistic regression (Table 1). The three-marker panel selected by logistic regression shared two markers in common with the MSS panel, and nearly all the samples were classified equivalently by MSS and logistic regression (data not shown). These analyses show that the MSS method can produce robust multi-marker panels that have consistent performance in cross validation and training set/test set analyses.

### Subclasses defined by marker states

**Figure 5:**
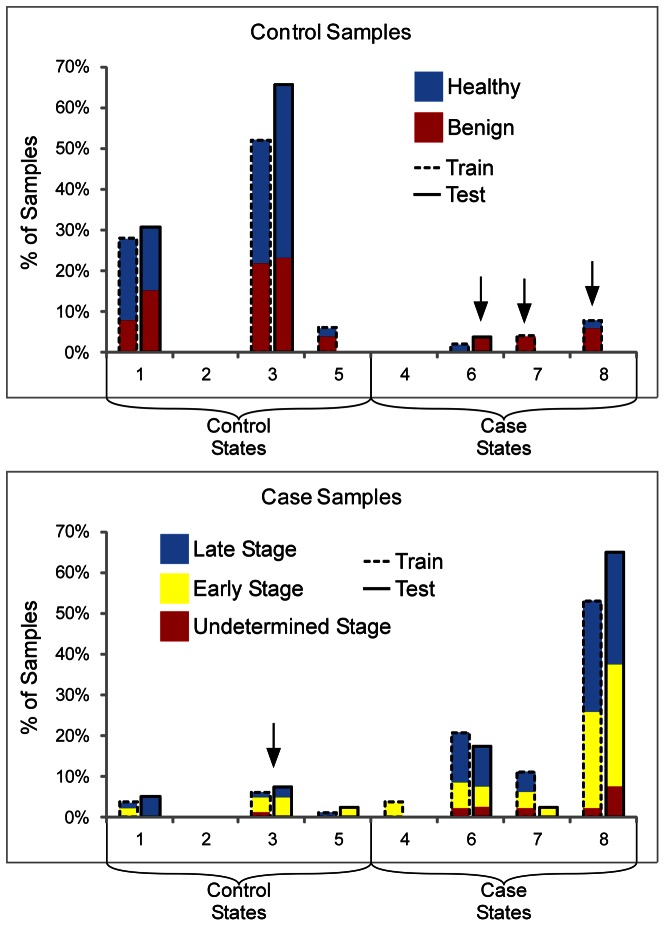
Composition of the states in the training and test sets. The percentage of control samples (top panel) and case samples (bottom panel) in each state is shown for both the training (dashed columns) and test sets. Consistency between the training and test sets in the relative occupancy between states is shown. The colors of the bars indicate the composition of the subjects within each state. The arrows indicate a high proportion of patients with benign disease (pancreatitis) in the case states (top panel) and a high proportion of early-stage cancer patients in the control state (bottom panel).

MSS enables a visualization of the marker states within the case and control samples and the contributions of each marker to the classifications, which may give insights into subclasses of patients based on marker states. A view of the marker states of each sample shows the relative proportions of the states (Figs. 3B and 4B). Among the control samples, most of the true negatives were in state 3 and state 1, with a small number in state 5, and the few false positives were in state 8, state 7, and state 6. The true positive samples were mainly in states 8 and 6, with a smaller number in states 7 and 4, while the false negative samples were in states 3, 1, and 5 (Figs. 3B and 4B). The relative occupancy of the states was similar between the training and test sets (Fig. 5), which suggests that these states would be consistent over a larger population.

We further tested the significance of these states by looking at the relative proportion of different sample types within each state. The control samples comprised both healthy subjects and patients with pancreatitis. We found that the control samples occupying the case states (the false positive samples) were predominantly from pancreatitis patients (Fig. 5), which is consistent with pancreatitis patients displaying more similarities to cancer patients than healthy people. In a similar way, the false negative samples primarily were from early-stage patients (stages I and II), rather than late-stage patients, consistent with an expected greater similarity of those patients to healthy people. Furthermore, we observed that state 6 seemed to have a higher percentage of late-stage cancer relative to state 8, as did state 1 relative to state 3. State 6 (1,0,1) differs from state 8 (1,1,1) in that the Blood group A-Blood group B marker is low, similar to the difference between state 1 (0,0,0) and state 3 (0,1,0). The proportions were observed in both the training and test sets, although their significance would have to been determined in larger studies. The differences in composition between states demonstrate the potential for using MSS to identify molecular subclasses with distinct phenotypic characteristics.

## Discussion

New biomarkers are needed in a wide range of applications. Because of the diversity between people and the possibility of subclasses of disease, any single biomarker may not have the performance needed to be clinically effective. The use of multiple markers together in a panel is a good approach for dealing with patient and disease diversity. Here we present a new method for forming biomarker panels. Marker State Space is built on the concepts that a set of all possible relationships (the state space) exists among a given set of markers, and that certain states are characteristic of disease. If we can accurately represent the underlying biological relationships across cases, robust biomarker performance should result. The method could be particularly valuable when the markers that define one subgroup are different from those that define another subgroup, or when the patterns of expression among a common set of markers are different between subgroups. MSS handles such diversity through the definition of distinct and independent states among patients.

We demonstrated that MSS can identify markers panels with robust performance in both cross validation and training set-test set analyses. These analyses showed that the selected panels were not “over fit,” or simply descriptive of only the training set data. The performance was similar to other methods of developing multi-marker classifiers. The fact that performance was similar between all the methods tested (Table 1) likely reflects the facts that the sample size is not large enough to reveal true differences in performance and that a limited amount of information was contained in these markers. The CA 19-9 marker, the current best individual marker for pancreatic cancer [33], dominated the classification, and the additional markers added a small amount of accuracy to CA 19-9, similar to previous research [30]. It is likely that no classification method will be best in every application; the optimal classification method will depend on the relationships in the data. The advantage of one method over another may appear only in the analysis of very large datasets. Here we wanted to test whether MSS could provide robust classification that was comparable to other methods and whether MSS had other practical advantages, as discussed below. The data show that MSS can provide classification accuracy on par with other, established methods, and that it may have advantages in certain settings.

The classification method bears resemblance to other methods such as recursive partitioning and k-nearest neighbors, but with some important distinctions. Recursive partitioning defines “case” states and “control” states based on discreet marker patterns, but because of the sequential searching using only the best marker at each division, the method may miss panels of markers that individually do not provide discrimination information but rather only in combination with specific other markers. K-nearest neighbors shares with MSS the classification of samples based on a vector of markers. The k parameter defines the size of the subset of nearest neighbors to which an unknown sample is matched, which presents a difficulty with unknown sizes of subclasses. MSS makes no assumption about nearest neighbors (a subclass of only one sample could be found). Another important characteristic of MSS relative to other classification methods is its suitability to clinical implementation, as it limits the number of markers and uses simple classification rules that do not require complex calculations. The use of small panels limits the cost of the test and reduces technical complexity, so that any laboratory that currently runs single assays could adopt a three-marker panel. The thresholding of each marker measurement and classification of each sample based on the pattern of three markers could be immediately implemented without special computation.

The ability of MSS to identify patient subclasses based on marker states was shown in two ways. First, the relative occupancy of the marker states was consistent between the training and test sets (Fig. 5), suggesting that these states are a natural feature of the population of cancer patients, rather than a random observation. Second, we observed differences in patient composition between the states (Fig. 5), suggesting real biological or clinical differences between the states. The high proportion of control patients with benign pancreatic disease in the “case” states and the high proportion of early-stage cancer patients in the “control” states explained the origin of the false positive and false negative identifications, respectively. These subsets of patients may be fundamentally different from the correctly classified patients, and additional markers may be necessary for their proper classification. These types of analyses can be used to suggest molecular classifications of patient subgroups and guide strategies for further exploration of molecular characteristics.

The composition of the marker panels also gives information about the states. The panel selected here used two different assays for the CA 19-9 antigen. We previously showed that these two antibodies have slightly different and complementary specificities [32], similar to previous studies of antibodies directed against glycan epitopes [34], [35], so their use together in a single panel could be expected to be beneficial. These two markers were complemented by the detection of the blood group B epitope on proteins captured by an antibody against blood group A (signified Blood group A-Blood group B). Patients in state 4 or state 7 were elevated in this marker and in only one of the CA 19-9 markers. A relatively small number of patients were in state 4 or 7 (Fig. 5), especially in the test set, but the appearance of these states suggest that certain patients who do not fully elevate the CA 19-9 antigen produce elevated levels of blood group A or blood group B antigens. The ABO blood group antigens are carbohydrate structures that are related to the carbohydrate structure defined by the CA 19-9 antigen [36], so a biosynthetic shift from one to the other in certain cancer patients would be possible. Additional studies would be required to study the relationship between these markers and the sources of variation between the patients.

The program and method could be further developed in various ways. The software could be expanded to allow more detail in the analysis. For example, instead of using one threshold per marker to convert each measurement to a 0 or 1, two thresholds could be used, resulting in three levels (0, 1, or 2) for each measurement. Such a modification might allow better modeling of situations in which three biologically-driven levels of a marker might exist across patients, such as abnormally low, moderate, and abnormally high levels. In addition, the software could be modified to select searches for panels including 3, 4, 5, or 6 markers, which could allow for greater diversity in the markers that are selected across patient states. The optimal panel size could be determined using methods that have been worked out for other classification algorithms, such as comparing change in performance from training set to test set as panel size is increased. The continued use of the method by additional researchers (the software is available upon request) will provide more information about its performance for biomarker research and reveal areas for further development.

A limitation to implementing these approaches in the current version of the software is the use of comprehensive searching to find the best panel. Comprehensive searching allowed us to identify the most robust panel for this data set, which represents an advantage over approaches that trim data in a stepwise process, as with recursive partitioning [16]. However, the run time for comprehensive searches increases exponentially with the increase in panel size or marker levels. The high search time might make comprehensive searching impractical for large panels or for datasets with many potential markers, as with gene expression data. In this work we used small panels to reduce the chance of overfitting, but to test the value of larger panels, alternate strategies will be needed, such as developing an analytical understanding of how to limit the search space or sampling the search space. Sampling would cover the range of possible panels but not comprehensively, thus running the risk of not finding the true optimum. Limitations to the search space could be imposed to allow comprehensive searching under constrained conditions. For example, we could search only a subset of all possible states, such as those with relatively simple classification rules. We demonstrated simplified classification rules in this work (Fig. 3A), reducing the 8 possible states for a 3-marker panel down to 6 states. In a similar way, the 16 possible states for a 4-marker panel could be reduced to a more manageable number of simplified states. It may be possible to first find the simplified states by comprehensively searching among smaller panels, and then to search for combinations among more markers using just the simplified states. These strategies will be explored as we further develop the use of the method.

Another limitation of the current implementation of MSS in comparison to some methods is that it cannot handle missing values, because the classification of each sample requires a complete, defined state. Approaches for dealing with missing data [37] potentially could be developed for MSS, such as by identifying partial states that are consistent with the observations and predicting the likelihood of each complete state.

In summary, we anticipate that the MSS method will provide a good complement to the existing approaches for developing biomarker panels. The method may allow for an accurate handling of subgroups within a population that have completely divergent marker profiles, which may result in improved performance over very large sample sizes. The simple computational process, involving the conversion of each measurement to binary values followed by the classification of each sample based on the marker state, should facilitate implementation in a wide range of settings. Future applications of the method could incorporate diverse data types, such as genotypes or clinical features. Such data types may be particularly appropriate for the MSS method because they indicate clear subgroups in populations.

## Supporting Information

Figure S1 **Antibody arrays with glycan detection.** In this example, three identical arrays containing three different antibodies (AB1, AB2, and AB3) are incubated with plasma, and proteins and captured according to the specificities of each antibody. Each array is probed with a different detection antibody, AB4, AB5, or AB6. The detection antibodies target specific glycan structures attached to the proteins. The detection antibodies are tagged (yellow circle) to allow measurements of their binding at each capture antibody. Nine different combinations of capture antibodies and detection antibodies are achieved
(PNG)

Table S1 **Antibodies and reagents used in the microarray experiments.**
(DOCX)
