# Supplementary figures and images for: The Marker State Space (MSS) Method for Classifying Clinical Samples
Source: PLoS One. 2013 Jun 4;8(6):e65905. doi: 10.1371/journal.pone.0065905 (PMC3672150; doi:10.1371/journal.pone.0065905)

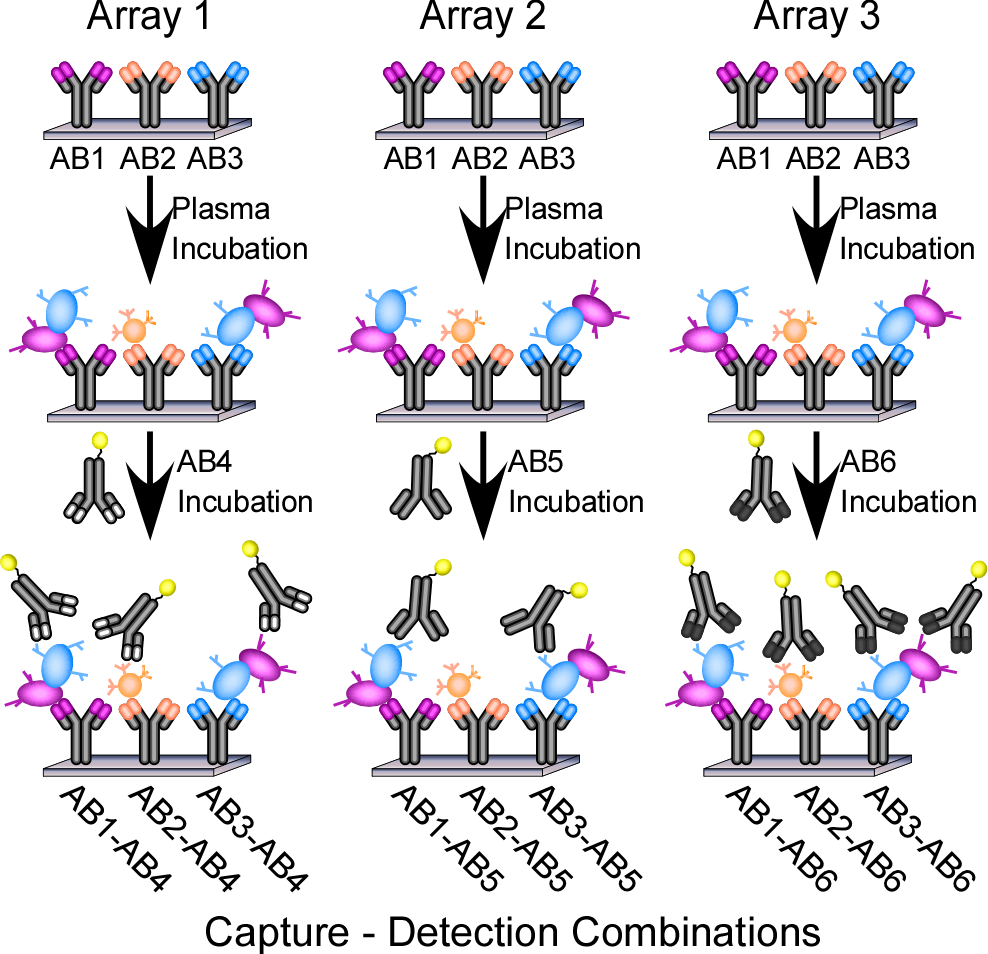

Supplement: Figure S1 — Antibody arrays with glycan detection. In this example, three identical arrays containing three different antibodies (AB1, AB2, and AB3) are incubated with plasma, and proteins and captured according to the specificities of each antibody. Each array is probed with a different detection antibody, AB4, AB5, or AB6. The detection antibodies target specific glycan structures attached to the proteins. The detection antibodies are tagged (yellow circle) to allow measurements of their binding at each capture antibody. Nine different combinations of capture antibodies and detection antibodies are achieved (PNG) [file pone.0065905.s001.png]
