# Supplementary material for: The Marker State Space (MSS) Method for Classifying Clinical Samples
Source: PLoS One. 2013 Jun 4;8(6):e65905. doi: 10.1371/journal.pone.0065905 (PMC3672150; doi:10.1371/journal.pone.0065905)
Supplement: Table S1 — Antibodies and reagents used in the microarray experiments. (DOCX) [file pone.0065905.s002.docx]

**Table S1. Antibodies and reagents used in the microarray experiments.** *Glycoconjugates printed on the arrays.

| Name | Species | Obtained from | Clone # | Catalog # |
| --- | --- | --- | --- | --- |
| Anti DUPAN-2 | Mouse | Glycotech | - | 11-010 |
| Anti Maltose Binding Protein (neg. control) | Mouse | Dr. Brian Cao, VARI | 5F12 | - |
| Anti Glutathione S-transferase (neg. control) | Mouse | Dr. Brian Cao, VARI | 8C1 | - |
| Anti *Bacillus anthracis* Lethal Factor (neg. control) | Mouse | Dr. Brian Cao, VARI | - | - |
| Anti Blood Group H | Mouse | Abcam | 87-N | ab24222 |
| Anti Blood Group Lewis b | Mouse | Thermo Scientific | 2-25LE | MA1-19346 |
| Anti Blood Group Sialyl Lewis x | Mouse | USBiological | 9L648 | S1013-51B |
| Anti Blood Group A | Mouse | Abcam | 9A | ab20131 |
| Anti Blood Group B | Mouse | Abcam | Z5H-2 | ab24224 |
| Anti Mucin 16 (Ab2) | Mouse | Novus Biologicals | X306 | NB120-10032 |
| Anti Mucin 1 (Ab1) | Mouse | Neoclone | 614D | - |
| Anti Blood Group Lewis x | Mouse | Abcam | P12 | ab3358 |
| Anti Blood Group Lewis a | Mouse | Abcam | 7LE | ab3967 |
| Anti Carcinoembryonic Antigen (CEA) | Mouse | USBiological | 2.Q.397 | C1299-94 |
| Anti Von Willebrand factor | Rabbit | DAKO | polyclonal | A0082 |
| Anti Mucin 5AC (Ab1) | Mouse | AbD Serotec | 45M1 | 1695-0128 |
| Anti Mucin 5AC (Ab2) | Mouse | Thermo Scientific | 2-11M1 | MA1-35704 |
| Anti Mucin 3A | Mouse | Lifespan Biosciences | Not Listed | LS-C16658 |
| Anti Mucin 3 | Mouse | Abcam | M3.1 | ab24068 |
| Anti Mucin 2 | Mouse | Abcam | 994/152 | ab22712 |
| Anti Mucin 16 (Ab1) | Mouse | Abcam | X325 | ab10033 |
| Anti Mucin 1 (Ab2) | Mouse | GeneTex | CM1 | GTX10114 |
| Anti Insulin-like Growth Factor 1 | Goat | R&D Systems | polyclonal | AF-291-NA |
| Anti Human Milk Fat Globule 1 | Mouse | Thermo Scientific | EDM45 | MS-512-P1 |
| Anti HA Tag (neg. control) | Mouse | Dr. Brian Cao, VARI | polyclonal | - |
| Anti Endorepellin | Goat | R&D Systems | polyclonal | AF2364 |
| Anti CEACAM6 | Mouse | Santa Cruz | By114 | sc-20059 |
| Anti Cancer Antigen 19-9 (Ab2) | Mouse | Abcam | 121SLE | ab3982 |
| Anti Cancer Antigen 19-9 (Ab1) | Mouse | USBiological | 9L426 | C0075-03A |
| Anti Bradykinin | Rabbit | AbD Serotec | polyclonal | 0100-0443 |
| Anti *Bacillus anthracis* Protective Antigen (neg. control) | Mouse | Dr. Brian Cao, VARI | 10F5 | - |
| Galα1,3Galβ1,4GlcNAc-Sp-BSA | * | Vector Labs Inc. | - | NGP0334 |
| N-acetyllactosamine-BSA | * | Vector Labs Inc. | - | NGP1201 |
| 3'-Sialyl-N-acetyllactosamine-BSA | * | Vector Labs Inc. | - | NGP1301 |
| Lacto-N-fucopentanose III-BSA | * | Vector Labs Inc. | - | NGP0502 |
| Lewis x-BSA | * | Vector Labs Inc. | - | NGP0302 |
| Neu5Acα2,3Galβ1,4(Fucα1,3)Glc-sp-BSA | * | Vector Labs Inc. | - | NGP0405 |
